# Inter-brain coupling tracks emotional co-regulation

**DOI:** 10.3758/s13415-026-01455-9

**Published:** 2026-05-23

**Authors:** Yafeng Pan, Franck Porteous, David Rosenbaum, Andreas Fallgatter, Anne-Christine Ehlis, Sander L. Koole, Suzanne Dikker

**Affiliations:** 1 Department of Psychology and Behavioral Sciences, Zhejiang University, Hangzhou, China; 2 The State Key Lab of Brain-Machine Intelligence, Zhejiang University, Hangzhou, China; 3 Department of Clinical Psychology, Vrije Universiteit Amsterdam, Amsterdam, the Netherlands; 4 Department of Psychiatry and Psychotherapy, Tübingen Center for Mental Health (TüCMH), University Hospital of Tuebingen, Tuebingen, Germany; 5 German Center for Mental Health (DZPG), Partner Site, Tübingen, Germany; 6 Department of Experimental Psychology, Ghent University, Ghent, Belgium; 7 Zhejiang Key Laboratory of Neurocognitive Development and Mental Health, Zhejiang University, Hangzhou, China; 8 Department of Psychology, New York University, New York, USA

**Keywords:** Inter-brain coupling, Emotional sharing, FNIRS hyperscanning, Mood, Empathy

## Abstract

When we have a negative emotional experience, we often recount this experience to others. Such emotional sharing plays a key role in building and maintaining interpersonal relationships, and is a vital component of most psychotherapies. Yet, while research shows the importance of social relationships for brain health, the neural underpinnings of emotional sharing remain largely unknown. Here, we asked whether successful processing and regulation of negative emotions can be linked to shared brain responses between dyads during and after emotional sharing. Participants watched videos eliciting either negative or neutral emotions, after which they shared their feelings about these videos with a friend. We simultaneously recorded the brain activity of both friends using functional near-infrared spectroscopy (fNIRS) and compared inter-brain coupling within sharer-listener dyads before, during, and after sharing sessions. We found that shifts in inter-brain coupling were associated with changes in mood. Specifically, an increase in inter-brain coupling after recounting a video that elicited negative emotions was associated with reduced anger and dejection in listeners and increased vigor in sharers. These findings suggest that inter-brain coupling facilitates the co-regulation of negative emotions, and thereby maintaining a healthy homeostatic balance. This knowledge holds potential relevance for informing psychotherapeutic interventions.

**Supplementary Information:**

The online version contains supplementary material available at https://doi.org/10.3758/s13415-026-01455-9.

## Introduction

Whenever people experience an emotional event, they are inclined to share their emotions with others. People report sharing their emotions with a partner 80% to 95% of the time after experiencing an emotional episode (Rimé et al., 1998; Singh-Manoux & Finkenauer, 2001; for a review, please see Rimé, 2009). Emotional sharing, defined as the process of mutually expressing and exchanging emotions, plays a vital role in strengthening interpersonal relationships (Cheng et al., 2024; Duprez et al., 2015; Rimé et al., 2020). Moreover, emotional sharing is a key component of virtually all mainstream psychotherapies, during which patients have to share their emotions with their therapists (Koole & Tschacher, 2016). The absence of emotional sharing can lead to neurobehavioral challenges (e.g., accelerated cortical thinning, social relationship problems; Rimé et al., 2020; Corrigan et al., 2024). These findings underscore the importance of understanding the neural processes underlying the social sharing of emotions. Yet, not much is known about these processes, especially at the interpersonal level.

A growing body of research indicates that processes of interpersonal coordination, such as successful cooperation (Cui et al., 2012; Pan et al., 2017), communication (Jiang et al., 2012, 2015), and emotional disclosure (Cheng et al., 2024), are associated with inter-brain coupling (IBC). Most existing research has focused on IBC during interactions and its associations with social outcomes. However, little is known about the inter-brain dynamics that occur after such interactions (Reinero et al., 2021; Shamay-Tsoory et al., 2024). The concept of sustained IBC after social interaction is supported by the need for mechanisms that allow people to achieve, maintain, or recover synchrony to reach common goals, even when social interactions are interrupted (Khalil et al., 2022). Recent imaging studies have observed a significant increase in IBC after dyadic musical interaction (Khalil et al., 2022), after teaching and social learning (Zheng et al., 2020), and after face-to-face movement synchronization (Shamay-Tsoory et al., 2024). These increased IBC following the offset of social interaction has been reported to reflect neuroplasticity and holds functional significance (Shamay-Tsoory, 2022). For instance, using functional near-infrared spectroscopy (fNIRS) hyperscanning (for details about this technique, please see Cui et al., 2012), Shamay-Tsoory et al. (2024) found that post-interaction IBC in the right inferior frontal gyrus was higher than pre-interaction (and even higher than interaction), and predicted levels of motivation to connect socially. A limitation of these studies is that they focus on IBC during interaction and after interaction separately, leaving possible connections between IBC during and after interaction underexplored. As a result, it remains unclear whether post-interaction IBC reflects a residual byproduct of prior synchrony or a sustained interpersonal process that emerges from ongoing regulation between partners. Addressing this gap requires an explicit temporal framework that bridges neural dynamics during and after social engagement. Also, the relationship between the development of IBC patterns and mood changes associated with emotional sharing remains unclear.

Emotional sharing provides a rich context wherein these dynamics and relationships can be examined. Rather than reflecting synchrony alone, emotional sharing relies on effective “co-regulation” (Butler & Randall, 2013), the dynamic process by which interactants mutually adjust their affective states to avoid emotional escalation and achieve mood balance. Individuals in close relationships, such as friendships (Zhu et al., 2025), often exhibit increased sensitivity to their partners’ emotions, facilitating reciprocal adjustments in emotional responses (Avnor et al., 2025). Importantly, co-regulation goes beyond simply mirroring each other’s emotional states, which can lead to escalating arousal levels, or “co-dysregulation” (Reed et al., 2015). Rather, co-regulation aims to keep the emotional arousal of both individuals within a healthy homeostatic balance (Timmons et al., 2015). For example, during emotional sharing, if a sharer gets in a negative mood, it is less effective for the listener to mirror that negativity. Instead, it is more constructive for the listener to respond in complementary ways, helping both individuals restore their emotional equilibrium. Despite these insights, the ways in which co-regulation manifests in mood states and IBC patterns during and following emotional sharing remains underexplored. Understanding these temporal dynamics is essential, as emotional sharing involves continuous co-regulation of affect between partners (both while emotions are exchanged and as they subside afterward). Bridging the gap between IBC during and after interaction may thus provide critical insight into how shared neural activity supports successful emotional co-regulation in social contexts.

The present study aims to address these limitations by examining IBC between individuals during and after emotional sharing by using the fNIRS hyperscanning approach. In a validated emotional sharing task (Nils & Rimé, 2012), a “sharer” watched a video eliciting negative or neutral emotions, and then shared their emotions with a “listener”, who was instructed to respond with socio-affective understanding and cognitive reframing (i.e., expressing understanding and reframing negative experiences into positive ones). Thus, while the interaction is embedded within an emotional sharing context, the design specifically facilitates regulatory engagement by the listener, enabling examination of neural processes supporting interpersonal co-regulation. We computed IBC between the sharer and the listener. We hypothesized that IBC would emerge in emotional sharing, even after interaction. We also expected that fluctuations in IBC would be associated with mood changes following emotional sharing. Note that the effects of emotional sharing on mood are complex: Prior research has reported mixed findings, with some studies observing improvements in mood and others highlighting increased negative mood states (see the dual mode theory for a discussion on this complexity; Rimé, 2009). By employing hyperscanning, we aim to shed further light on the nuanced relationship between emotional sharing and mood changes. Our regions of interest include bilateral fronto-temporo-parietal regions (including inferior frontal cortex and temporo-parietal junction), which have been previously associated with social interactions including emotional disclosure using fNIRS hyperscanning (Cheng et al., 2024; Kruppa et al., 2021; Pan et al., 2017; Reindl et al., 2018; Shamay-Tsoory et al., 2024; Tang et al., 2015). Although emotional contagion and co-regulation are sometimes used interchangeably, they reflect complementary (not necessarily competing) interpersonal mechanisms. Emotional contagion refers to the relatively automatic convergence of affective states between individuals, often resulting in shared increases in emotional intensity. In contrast, co-regulation involves adaptive, and frequently complementary, adjustments that help partners maintain or restore affective balance. The present design allows us to test these accounts by examining whether mood and IBC patterns are consistent with mirroring or with dynamic modulation across interaction phases.

## Method

### Participants

This study is part of the Rhythm of Relating research project (NWO, 406.18.GO.024), which sought to explore how emotional sharing arises from interpersonal synchrony and how this can lead to long-term emotion-regulatory benefits. Forty participants (36 females, 4 males; *M*_age_ = 21.73, *SD*_age_ = 3.21) were recruited at the Eberhard Karls University of Tübingen and participated in the experiment, resulting in the formation of twenty same-sex friend dyads. This sample size was determined based on prior hyperscanning studies investigating IBC during emotion-related social interaction in close relationships, which typically included 17–20 dyads to achieve adequate statistical power for detecting IBC (e.g., Goldstein et al., 2018; Zhang et al., 2023). Participants were only allowed to participate if they had been friends for at least six months. Thirty-seven participants identified as German or European (92.5%), one as Asian (2.5%), one as Kurdish, and one as Turkish. Participants were randomly assigned to begin with either the emotional sharing or the neutral sharing condition. Neuroimaging data from two dyads were excluded due to excessive artifacts and poor signal-to-noise ratio, leaving eighteen dyads with usable data for subsequent analyses. The methods and procedures of this study adhered to the Declaration of Helsinki. The ethics committee at the Faculty of Medicine of University of Tübingen approved this study (603/2016BO2). All participants were provided with detailed explanations of all procedures and potential risks, both verbally and in writing. They were also informed of their right to withdraw from the experiment at any time without providing a reason and without facing any consequences. Written informed consent was obtained from all participants. Participants received monetary compensation for their participation.

### Experimental tasks and procedures

**Fig. 1:**
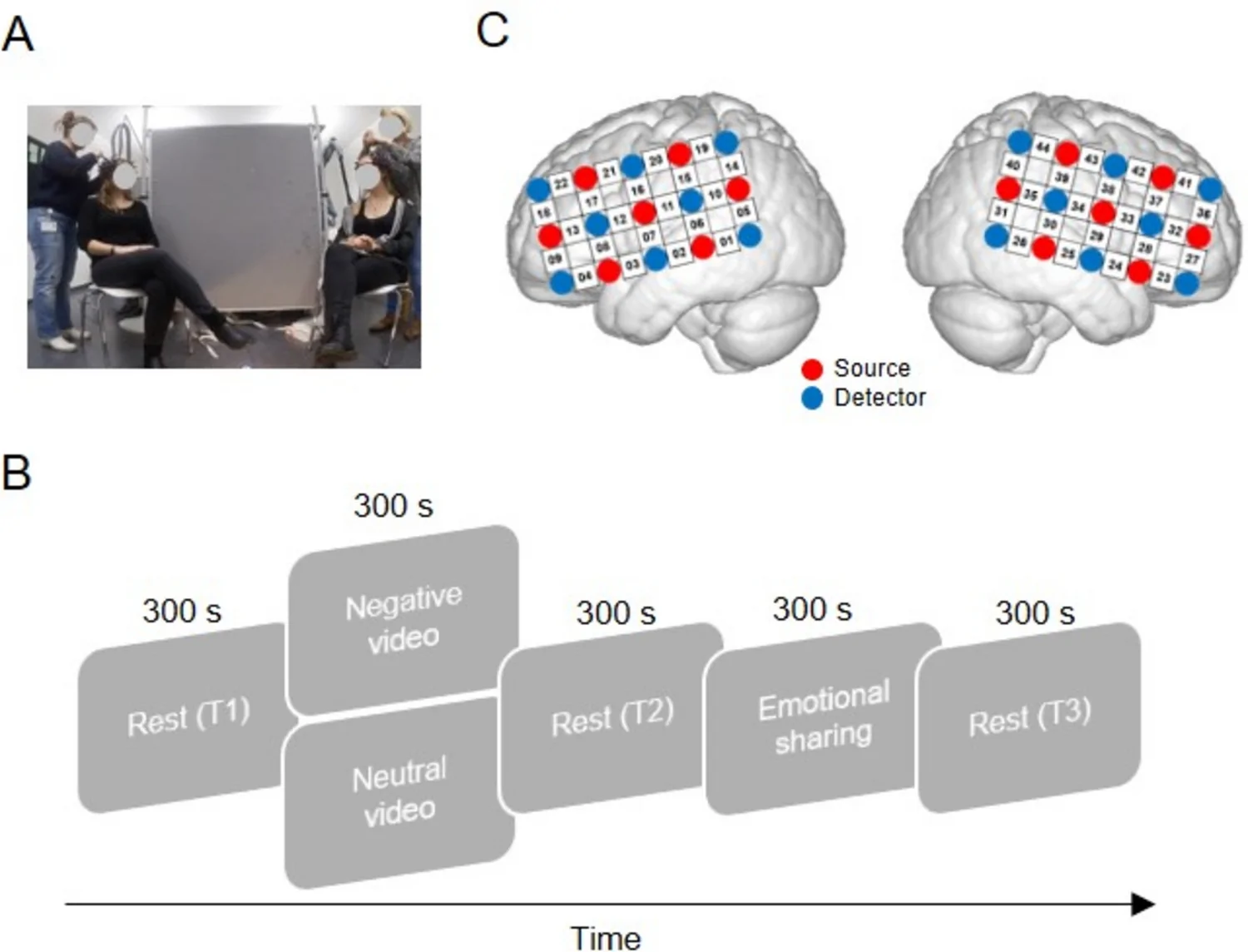
Experimental setting, procedure, and probe location. (**A**) During emotional sharing, the sharer and listener sat face-to-face. (**B**) The experiment consists of two blocks: a 5-min video (either negative or neutral) watching block and a 5-min emotional sharing block, with a 5-min resting period before and after each block. These resting periods correspond to three time points (T1-T3) for measuring participants' mood changes. T2 and T3 also correspond to the pre-sharing and post-sharing stages. (**C**) The fNIRS optode probe set was positioned over the participant’s bilateral fronto-temporo-parietal regions

The experiment consisted of two sessions, each corresponding to one of two experimental conditions (emotional sharing vs. neutral sharing), separated by a week. Participants were randomly assigned to one of two roles: sharer or listener (Fig. 1A). Each session included a 5-min *video-watching* block, followed by a 5-min *emotional sharing* block, with a 5-min resting period before and after each block (Fig. 1B). These resting periods correspond to three time points (T1-T3) for measuring participants' mood changes (see next section for details). T2 and T3 also correspond to the pre-sharing and post-sharing stages.

During the video-watching block, the sharer watched either an emotional or a neutral 5-min video consisting of documentary clips about human nature. The negative emotion-inducing video contained scenes portraying human suffering and cruelty (e.g., the killing of seals in Canada for their fur and starving children in Africa) and was adapted from a stimulus set validated in a previous emotional sharing study, which confirmed its effectiveness in eliciting negative affective states (Koole & Tschacher, 2016). The neutral video contained neutral imagery about human nature, featuring two men from Siberia building a boat from a tree trunk, the discovery of a secret room in a famous Egyptian tomb, and a man crafting globes of Earth and other planets by hand. These clips were selected to match the negative videos in duration, narrative style, and presence of social content, while avoiding scenes likely to elicit strong emotional responses. This matching ensured that differences between conditions could be attributed to emotional valence rather than other perceptual or cognitive features. While the sharer watched the video, the listener received written instructions on having a conversation with their counterpart for approximately five minutes.

During the emotional sharing block, the listener was given suggestions for questioning their counterpart, such as: "What did you see in the video clips?" "What did you think of the video clips?" "Do you consider that viewing such sequences could be useful?". The listener was informed about the video content while the sharer watched the video and was instructed to let the conversation flow naturally. In the emotional sharing condition, the instructions were more detailed, requesting the listener to take a socio-affective and cognitive reframing approach, i.e., expressing understanding and reframing negative experiences into positive ones.

To ensure that the sharing process was comparable across conditions (emotional vs. neutral), the instructions emphasized maintaining similar conversational length and engagement. The experimenters monitored the interactions in real time and instructed listeners to keep the conversation flowing naturally while following the guidelines. This structured yet flexible approach allowed the listener to actively pay attention to the sharer’s emotions while keeping interaction patterns broadly consistent across conditions, ensuring that observed differences in IBC could be attributed to emotional valence rather than differences in interaction magnitude or style. Nonverbal behaviors, particularly body movements, were monitored and recorded throughout the sharing stage. We quantified movement synchrony between dyad members in both conditions using a motion energy analysis approach (Ramseyer, 2020; see Supplementary Materials). No significant difference in movement synchrony was observed between conditions: emotional sharing (0.14 ± 0.03) and neutral sharing (0.15 ± 0.03) were comparable, paired *t* = 1.58, *p* = 0.14. This indicates that any observed differences in IBC are unlikely to be driven by differences in dyadic movement.

### Subjective measurements

Before the experiment, participants completed self-report measures of empathy and mood. After each block, participants also reported their subjective mood.

#### Empathy

We used the German version of the Interpersonal Reactivity Index (IRI) (Davis, 1983), which measures four independent components of empathy: (a) perspective-taking (PT; the tendency to spontaneously adopt the psychological point of view of others), (b) empathic concern (EC; the tendency to experience feelings of warmth, compassion, and concern for others undergoing negative experiences), (c) fantasy (FS; empathy with fictional characters in books, movies, and plays), and (d) personal distress (PD; self-focused feelings in the presence of others suffering). The German version of the IRI was rated on a scale of 1 (not at all) to 5 (very much), with higher scores indicating greater empathic skills. After reverse-scoring, items were averaged per subscale. Cronbach’s α for the PT, EC, FS, and PD scales were 0.77, 0.71, 0.76, and 0.81, respectively.

#### Mood

Participants rated their mood using a brief German version of the Profile of Mood States (POMS) (Albani et al., 2005). This version of the POMS includes 35 emotional descriptors rated from 0 (not feeling the emotion at all) to 6 (strongly feeling the emotion). These descriptors are divided into four domains of negative mood: "dejection" (e.g., feeling sad, helpless, miserable), "vigor" (e.g., energetic, lively, active), "fatigue" (e.g., tired, exhausted, listless), and "anger" (e.g., angry, irritated, bad-tempered). Each subscale consists of 7 items, except "dejection," which has 14 items. The listener rated the POMS measure twice, before (T1, Cronbach’s α = 0.60) and after the experiment (T3, Cronbach’s α = 0.62), while the sharers rated it three times, including after watching the video (T2, Cronbach’s α = 0.62). We performed a repeated-measure ANOVA on average self-reported mood for both sharers and listeners, with Time [T1 (vs. T2) vs. T3] and Condition (emotional sharing vs. neutral sharing) as two within-subject variables.

### fNIRS data acquisition

Throughout the experiment, hemodynamic signals of participants in each dyad were simultaneously recorded using two ETG-4000 systems (Hitachi Medical Corporation, Japan). The absorption of near-infrared light was measured at two wavelengths (695 and 830 nm). Raw intensity signals were recorded at a sampling frequency of 10 Hz. Each participant wore two "3 × 5" optode probe sets configured to measure 44 channels (7 sources and 8 detectors for each hemisphere, inter-optode distance = 30 mm; Fig. 1C). The correspondence between the NIRS channels and measurement points on the cerebral cortex was determined using the virtual registration method (Singh et al., 2005; Tsuzuki et al., 2007). Consistent with previous fNIRS studies (Cui et al., 2012; Pan et al., 2023), this study focused solely on HbO concentration due to its higher signal-to-noise ratio compared to HbR (Mahmoudzadeh et al., 2013), and its greater sensitivity as an indicator of regional cerebral blood flow in fNIRS measures (Hoshi, 2007). To ensure temporal synchrony across participants, all recording systems were synchronized through hardware triggers generated by the experimental presentation computer. The onset markers of each task phase (e.g., video watching, sharing, resting) were recorded simultaneously in both data streams. HbO signals were then temporally aligned according to these triggers before further analysis.

### fNIRS data analysis

#### Preprocessing

All analyses were performed using MATLAB 2019b, with standard functions and toolboxes. We applied Correlation-Based Signal Improvement (CBSI) to eliminate head motion artifacts (Cui et al., 2010). CBSI reduces noise by maximizing the negative correlation (assumed to be close to −1) between the HbO and HbR signals. Because CBSI enforces an anti-correlated structure between HbO and HbR, the corrected HbR signal becomes a linear transformation of the corrected HbO signal. Accordingly, after CBSI correction, HbR mirrors HbO, and statistical inferences based on HbO fully capture the information contained in the corrected signals (Tsz-lok Lee & Chan, 2024; Tsz‐lok Lee et al., 2024; Pan et al., 2023). Additionally, we employed a wavelet-based denoising approach to remove global physiological noise, such as blood pressure and respiration artifacts (Duan et al., 2018). This method involved automatically detecting the time–frequency points contaminated by global physiological noise using wavelet transform coherence. We then decomposed the fNIRS signal using wavelet transform and suppressed the wavelet energy of the contaminated time–frequency points. Preprocessing was performed using standard MATLAB scripts provided by the original method developers, including the *cbsi* (for CBSI; Cui et al., 2010) and *wtc_denoise* functions (for wavelet-based denoising; Duan et al., 2018).

#### Inter-brain coupling

We used Wavelet Transform Coherence (WTC) to assess IBC between sharers and listeners. WTC measures cross-correlation between time-series as a function of frequency and time (for more details, see Grinsted et al., 2004). Unlike static correlation measures, WTC decomposes signals into the time–frequency domain and estimates phase-consistent coupling between two time series, making it well suited for non-stationary fNIRS data and for capturing dynamic changes across interaction phases. This approach allows us to examine whether coupling during emotional sharing temporally relates to coupling observed afterward, rather than assuming stationarity across the entire recording period. We applied the *wcoherence* function in Matlab. IBC was calculated between each homologous channel (e.g., CH1-CH1, CH4-CH4). IBC for the pre-sharing (5 min), sharing (5 min), and post-sharing stages (5 min) was converted into Fisher-z statistics and time-averaged (Chang & Glover, 2010; Cui et al., 2012; Dai et al., 2018).

To determine the frequencies and regions of interest, we employed a cluster-based permutation test (Maris & Oostenveld, 2007). This nonparametric statistical test is well suited for fNIRS hyperscanning data because it effectively controls for multiple comparisons across channels and frequency bins while accounting for the spatial and temporal correlations inherent in neural signals. By clustering adjacent data points that show consistent effects, this method increases statistical sensitivity without inflating Type I error (Maris & Oostenveld, 2007; also see our recent application in fNIRS hyperscanning, Zhu et al., 2022; Pan et al., 2023). First, we conducted frequency-by-frequency (ranging from 0.01–1 Hz) and channel-by-channel (44 channels in total) paired-sample *t*-tests (emotional sharing vs. neutral sharing) on IBC for each stage (pre-sharing, sharing, and post-sharing). We directly compared the two conditions, as previous neuroimaging studies have shown that an active task condition provides a better baseline than rest conditions (Reindl et al., 2018; Stark & Squire, 2001). Second, we identified channels (44 in total) and frequencies (80 in total, ranging from 0.01 to 1 Hz) that showed a significant between-condition effect (emotional sharing vs. neutral sharing, *P* < 0.05). Third, clusters with neighboring channels (*N* ≥ 2) and/or neighboring frequency bins (*N* ≥ 2) were formed. The statistic for each cluster was computed by summing all the t values. We focused on the largest clusters with the highest absolute cluster statistics. Fourth, we repeated steps one through three 1000 times using permuted data. Permutations were constructed by randomly pairing one participant's data with data from another participant in a different condition. IBC was estimated for the randomized groups in the same way as for the real groups. Finally, significance levels (*P* < 0.05) were calculated by comparing the obtained cluster statistic from the real group with 1000 renditions of randomized groups. The “largest clusters” refer to those with the greatest cluster mass (i.e., the summed *t* statistics within a contiguous supra-threshold cluster) that survived cluster-level correction (permutation-based *P* < 0.05). These clusters were selected because cluster mass provides a robust and widely used index of effect magnitude in non-parametric cluster-based inference. Because cluster-based permutation approaches rely on non-parametric resampling and evaluate the summed statistics within significant clusters, conventional parametric effect size measures (e.g., η^2^) are not directly applicable. To ensure transparency and allow readers to gauge the magnitude of effects, we report the cluster-level statistics (i.e., cluster summed test statistics) and exact permutation-based *P*-values.

WTC assumes that meaningful coupling is reflected in temporally localized, frequency-specific coherence between oscillatory components. A limitation is that coherence estimates can be influenced by shared systemic physiological signals (e.g., respiration or cardiovascular rhythms). However, this limitation did not apply to our study. Specifically, the cluster-based permutation test identified two significant clusters (Fig. S1): Cluster 1, a negative IBC cluster at CH27 during sharing (frequencies 0.04–0.07 Hz), and Cluster 2, a positive IBC cluster at CH23 during post-sharing (frequencies 0.02–0.04 Hz). These frequencies allowed the removal of undesired signals in higher frequency bands, including those associated with Mayer waves (approx. 0.1 Hz), respiration (approx. 0.2–0.3 Hz), and cardiac pulsation (approx. 1 Hz). Computationally, the frequencies of interest correspond to periods of 14–25 s (Cluster 1) and 25–50 s (Cluster 2), time scales that are consistent with sustained, continuous utterances during emotional sharing. The IBC results yielded a *t* map for the bilateral hemispheres, generated with a spatial interpolation method and rendered over a standard brain template (ICBM 152 nonlinear asymmetric template) using the EasyTopo toolbox (Tian et al., 2013).

Based on studies suggesting a relationship between IBC and emotional co-regulation between partners, we expected that IBC was related to each participant’s self-reported mood. Hence, we conducted a series of Pearson correlation analyses to explore the relationship between IBC changes (IBC_post-sharing_—IBC_sharing_) and changes in POMS ratings (post-sharing vs. pre-sharing). Because emotional sharing is inherently relational, the dyad constitutes the primary unit of interaction in the present study. Accordingly, all IBC analyses are conducted at the dyadic level, quantifying shared neural dynamics between partners rather than treating individuals as independent units. Where analyses examine associations with mood, we explicitly distinguish dyad-level coupling metrics from individual-level affective reports, thereby separating interpersonal neural processes from intra-individual outcomes. This approach allows us to probe how shifts in IBC after emotional exchange relate to individual-level emotional adjustment.

Across statistical analyses, false discovery rate (FDR) correction was applied to control for multiple comparisons within each family of tests (Benjamini & Hochberg, 1995). For correlation analyses, we reported both uncorrected and corrected *P* values for transparency and to allow readers to fully evaluate the pattern of findings.

## Results

### Socially contagious negative mood

**Fig. 2:**
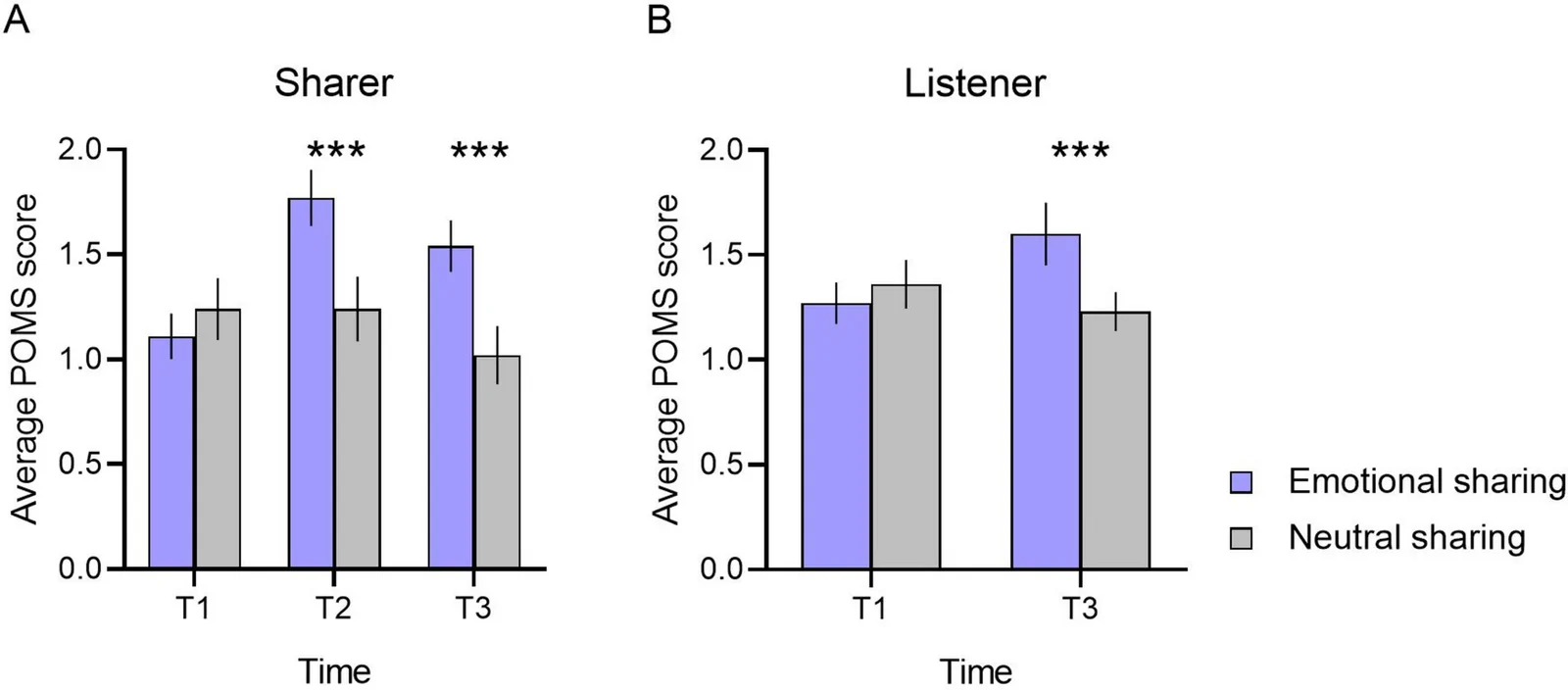
Mood changes as measured by the Profile of Mood States (POMS). (**A**) Average self-reported mood of the sharer at T1 (i.e., before video watching), T2 (i.e., after video watching and before emotional sharing), and T3 (i.e., after emotional sharing) for neutral and emotional sharing conditions. (**B**) Average self-reported mood of the listener at T1, and T3 for both conditions. Error bars denote standard errors of the mean. ***p < 0.001

For sharers, a repeated-measures ANOVA revealed a main effect of Time on mood, *F*_1,38_ = 15.12, *P* < 0.001, *η*^2^ = 0.29, and a significant interaction between Time and Sharing conditions, *F*_1,38_ = 19.32, *P* < 0.001, *η*^2^ = 0.34. There was no overall difference in mood between conditions at T1 (before video watching, Fig. 1B), indicating that the baseline mood levels were comparable across conditions. However, sharers reported an increase in negative mood state after watching the emotional video (*M* ± *SD*, 1.77 ± 0.60), compared to after the neutral video (1.24 ± 0.69), at T2 (i.e., after video watching and before emotional sharing, Fig. 1B). In addition, sharers reported more negative mood following emotional sharing in the emotional condition (1.54 ± 0.55) compared to the neutral condition (1.02 ± 0.62) at T3 (i.e., after emotional sharing). While there seemed to be a decreasing trend in negative mood from T2 to T3 (i.e., a general trend toward mood recovery), there was no significant difference observed between T2 and T3 in mood for either condition (Fig. 2A).

There was no significant difference in mood between conditions at T1, indicating that the baseline mood levels in listeners were comparable across conditions. The mood of listeners did not vary over time, *F*_1,38_ = 2.03, *P* = 0.16. However, there was a significant interaction between Time and Sharing conditions,* F*_1,38_ = 12.04, *P* < 0.001, *η*^2^ = 0.24. This interaction revealed that listeners reported a greater increase in negative mood at T3 (i.e., after emotional sharing, Fig. 1B) in the emotional sharing condition (1.60 ± 0.67) compared to the neutral sharing condition (1.23 ± 0.41) (Fig. 2B).

### IBC

**Fig. 3:**
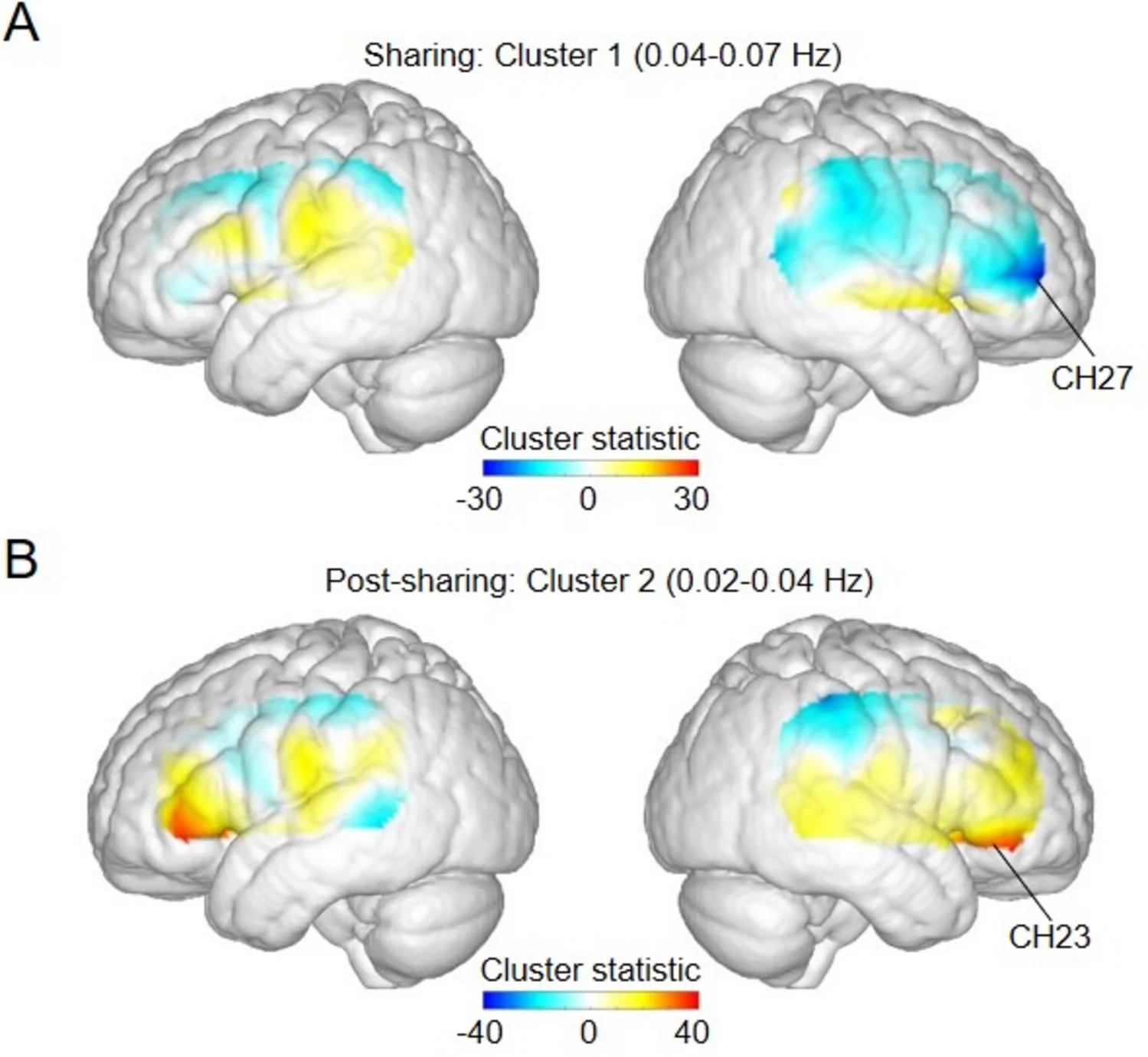
Inter-brain coupling (IBC). Cluster-based permutation tests (emotional sharing vs. neutral sharing) revealed 2 significant clusters: (**A**) a negative cluster at sharing, i.e., cluster 1 (CH27, frequencies 0.04–0.07 Hz), and (**B**) a positive cluster at post-sharing, i.e., cluster 2 (CH23, frequencies 0.02–0.04 Hz). Only the largest cluster with the highest absolute cluster statistic was retained for subsequent analyses. The cluster statistic denotes the sum of *t* values (paired-sample *t*-tests for emotional sharing vs. neutral sharing) within adjacent frequencies and/or channels

The cluster-based permutation analyses revealed distinct patterns across time points: (1) *Pre-sharing* (i.e., at T2): No significant IBC cluster was observed, indicating comparable IBC levels before emotional sharing. (2) *During sharing* (i.e., between T2 and T3): The emotional (vs. neutral) sharing condition elicited a significant negative IBC cluster at CH27. This effect was observed in the frequency range of 0.04 to 0.07 Hz (cluster statistics = −34.33, *P* < 0.001). (3) Post-sharing (i.e., at T3): The emotional (vs. neutral) sharing condition elicited a significant positive IBC cluster at CH23, within the frequency range of 0.02 to 0.04 Hz (cluster statistics = 41.43, *P* < 0.001). Both CH23 and CH 27 were located roughly in the right inferior frontal cortex (Fig. 3). The significant negative IBC cluster observed during sharing and the significant positive IBC cluster post-sharing illustrate the decrease and subsequent increase in IBC during emotional sharing compared to neutral sharing.

### Changes in IBC were associated with changes in mood

**Fig. 4:**
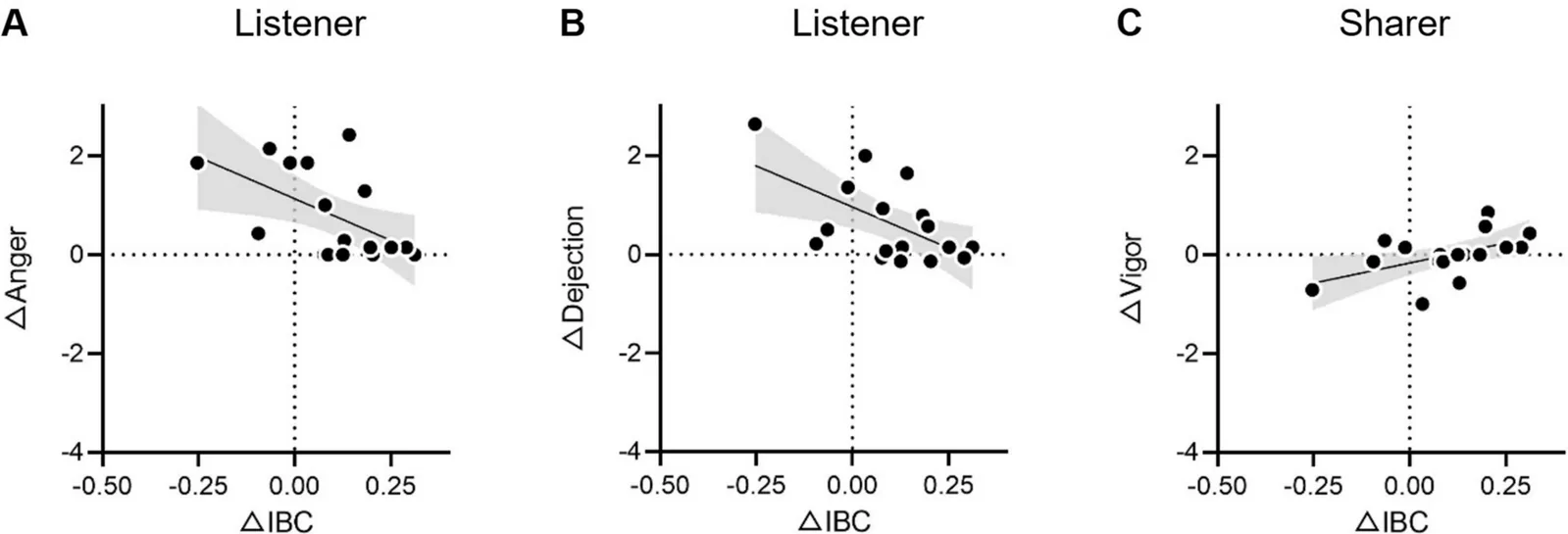
Changes in inter-brain coupling (IBC) were negatively correlated with changes in Anger (**A**) and Dejection (**B**) for listeners and positively associated with changes in Vigor for sharers (**C**) in the emotional sharing condition. △ = Post sharing minus sharing

Having identified IBC changes as a function of emotional sharing, we next sought to probe the relationship between IBC and negative moods. To capture temporal variations in IBC from interaction to post-interaction phases, we calculated difference scores (ΔIBC = IBC_post-sharing_—IBC_sharing_) and examined their associations with mood changes. We focused on the largest cluster, which provides a robust and widely used index of effect magnitude in non-parametric cluster-based inference, identified at each stage: Cluster 1 for IBC during sharing (CH27, 0.04–0.07 Hz) and Cluster 2 for IBC during post-sharing (CH23, 0.02–0.04 Hz). These clusters were then used to compute the IBC difference scores entered into the correlation analyses. This approach allowed us to assess how shifts in IBC after emotional exchange relate to subsequent emotional adjustment in each partner. In the emotional sharing condition, we observed that, for listeners, changes in IBC (i.e., IBC_post-sharing_—IBC_sharing_) showed negative correlations with changes in Anger, *r* = −0.55, uncorrected *P* = 0.023, corrected *P* = 0.046 (Fig. 4A), and changes in Dejection, *r* = −0.59, uncorrected *P* = 0.013, corrected *P* = 0.046 (Fig. 4B). In the sharers, we observed that changes in IBC were positively correlated with changes in Vigor *r* = 0.52, uncorrected *P* = 0.031, corrected *P* = 0.122 (Fig. 4C). No significant effects after multiple-comparison correction were observed in the neutral sharing condition. See Supplementary Fig. S2 for all comparisons.

### Complementary analyses

We performed two complementary analyses. First, to investigate the potential influence of passage of time on IBC (from interaction to post-interaction), we conducted a split-half analysis (Dikker et al., 2021). Specifically, we divided the entire emotional sharing process into two equal-length stages: early (first half, 2.5 min) versus late stage (second half, 2.5 min). Comparing IBC between these two stages, we found no significant difference (*t*_17_ = 0.05, *P* = 0.96). This result suggests that the observed effect is less likely to be confounded by the passage of time during the experiment.

Second, we then examined whether individual differences in self-reported empathy, assessed using the four subscales of the Interpersonal Reactivity Index, were associated with emotional sharing and could potentially explain the effect of IBC. However, we found no significant correlations between any of the four subscales and differential POMS ratings (*r*s < 0.31, *P*s > 0.22) or △IBC (*r*s < 0.27, *P*s > 0.27) in the emotional sharing condition. These findings suggest that IBC between sharers and listeners extends beyond participants' self-reported empathy, indicating that IBC measure captures an aspect of behavior that is not covered by traditional empathy measures.

## Discussion

In the present study, we used an fNIRS-based hyperscanning approach to investigate emotional sharing within sharer-listener dyads from a neural perspective. Behaviorally, we observed that sharers reported an initial increase in negative mood (perhaps because sharers had to talk about negative experiences) before emotional sharing, and a decreasing trend in negative mood (i.e., mood recovery) after emotional sharing; in contrast, listeners reported a significantly higher level of negative mood after sharers’ sharing, suggesting the social contagion nature of emotion. Neurally, we found that emotional sharing was characterized by a decrease in IBC, whereas post-sharing was associated with an increase in IBC. The changes in IBC were further correlated with changes in mood, highlighting its functional significance. Finally, we were able to distinguish emotional sharing from neutral sharing based on IBC. The classification performance improved when combining IBC during and after emotional sharing as features.

Behaviorally, sharers did not show a significant reduction (though there was a decreasing trend) in negative mood following emotional sharing, despite receiving socio-affective and cognitive reframing support from the listener. This aligns with prior work suggesting that the effects of emotional sharing are complex and may not immediately manifest as improved mood (Nils & Rimé, 2012; Rimé, 2009). Possible contributing factors include the short duration of the sharing period, individual differences in emotional reactivity, and limited sample size reducing statistical power. Interestingly, listeners’ mood worsened after interacting with sharers, consistent with previous evidence of vicarious distress during negative emotional sharing (López-Pérez & Ambrona, 2015). This finding might be due to emotional contagion, a phenomenon frequently noted in the emotional sharing literature (Butler & Randall, 2013; Christophe & Rimé, 1997). These findings highlight the dynamic nature of emotional co-regulation in social interactions.

We observed that IBC decreased during emotional sharing, and subsequently, increased after emotional sharing. A possible interpretation draws on a model of emotional co-regulation in close relationships (Butler & Randall, 2013), which proposes that co-regulation involves both emotional contagion (“morphostatic regulation”) and buffering. IBC likely reflects the process of emotional contagion. However, when the sharer experiences intense negative mood, emotional contagion could amplify the listener's negative affect to an excessive degree. To prevent this, listeners may shift toward buffering, thereby reducing their emotional contagion (and consequently IBC). As the sharer’s negative mood diminishes, emotional contagion can resume, enabling the listener to emotionally synchronize with the sharer once more.

Because brain signals were recorded simultaneously from interacting partners, observations within dyads are inherently non-independent. To address this, IBC was computed as a dyad-level metric, and all statistical tests on IBC treated the dyad as the unit of analysis. Comparisons across conditions and stages were conducted using within-dyad contrasts, thereby minimizing between-dyad variability. Nevertheless, this approach does not fully decompose shared versus individual-specific variance components, which should be considered when interpreting dyadic neural effects. Future studies with larger samples could extend this analytical structure by using dyad-level mixed-effects or multilevel models that explicitly account for shared variance within dyads while estimating individual-specific contributions.

As expected, fluctuations in IBC were associated with changes in mood. Specifically, IBC changes were positively correlated with changes in vigor (△Vigor) in the sharers and negatively correlated with changes in anger (△Anger) in the listeners. Our finding joins previous observations (Pan et al., 2018; Reinero et al., 2021; Zhang et al., 2018), consistent with the possibility that IBC is not merely a byproduct of interaction but has functional significance. Specifically, IBC was associated with mood recovery or improvement in sharers following emotional sharing and was linked to the reduction of negative mood in listeners, highlighting the benefits of emotional sharing (Luminet et al., 2000; Nils & Rimé, 2012; Rimé et al., 2020; Zech et al., 2004).

All IBC patterns observed in this study, whether the decreased IBC during emotional sharing or the increased IBC after sharing, implicated the inferior frontal cortex, particularly in the right hemisphere. The inferior frontal cortex is considered part of the mentalizing network (also known as the theory of mind network) (Fehlbaum et al., 2022; Monticelli et al., 2021) and aligns with the basic principles of the mirror neuron system (Rizzolatti & Sinigaglia, 2016). IBC in this region has been associated with a wide range of interactive activities, including social learning and teaching (Pan et al., 2018), face-to-face communication (Jiang et al., 2012), cooperative singing (Osaka et al., 2015), and game playing (Liu et al., 2016). Given its location, it is reasonable to infer that IBC in the inferior frontal cortex supports a range of cognitive functions, including action and emotion understanding (Rizzolatti & Sinigaglia, 2016), as well as the regulation of stress (Moazzami et al., 2020). The specificity of this region makes it an effective target for further intervention studies. Therefore, our findings are also relevant for therapeutic practices in populations with emotional challenges.

The present study has several limitations. First, we only measured mood recovery immediately after emotional sharing. Based on previous research, we expect that bonds between friends strengthen more from emotional sharing experiences compared to sharing neutral topics (Fischer & Manstead, 2008). Studies have also emphasized the importance of emotion for bonding (Collins & Miller, 1994; Peters & Kashima, 2007). However, these effects may take longer to manifest. Future studies should consider assessing rapport multiple times, including several days after an emotional versus neutral sharing experience. Second, the limited effect of cognitive reframing during emotional sharing on mood recovery may be due to the time limit of the sharing conversations in our study. Future studies investigating the benefits of cognitive reframing during emotional sharing should allow a more generous timeframe for this process. Relatedly, although listeners were instructed to engage in socio-affective understanding and cognitive reframing, we did not independently quantify the extent or quality of these behaviors across dyads. Future studies integrating hyperscanning with fine-grained behavioral coding or linguistic analysis could directly test whether variability in empathic validation or reframing predicts IBC dynamics and subsequent mood change. Importantly, our conclusions are limited by the small sample of the present study, making our findings preliminary pending formal replication. In particular, the observed associations between IBC and mood outcomes should be interpreted with caution. Another notable limitation of the present study is the strong gender imbalance in the sample (36 females, 4 males). Another notable limitation of the present study is the strong gender imbalance in the sample (36 females, 4 males). Although supplementary analyses excluding male participants yielded results consistent with the main findings, the small number of male participants precludes meaningful examination of sex differences or gender-related modulation of IBC and mood dynamics. Given prior evidence suggesting gender differences in affective sharing (Luo et al., 2015), future studies with more balanced and adequately powered samples will be necessary to determine whether the observed IBC patterns generalize across genders or are differentially expressed.

Another important avenue for future research is the adoption of more direct dyad-structured analytical approaches. Methods such as inter-subject representational similarity analysis (IS-RSA), widely used in fMRI research, could quantify how similarity structures within and between partners relate to behavioral or affective outcomes at a finer-grained level. Likewise, explicitly comparing dyads stratified by shared or mismatched characteristics (e.g., high–high, low–low, or mixed pairs based on empathy or related traits) may reveal moderating factors that shape IBC dynamics during emotional sharing. Such approaches would allow stronger inference about how dyadic similarity or complementarity influences both IBC and affective regulation. Implementing these designs will require larger samples to ensure adequate statistical power and stability of dyad-level estimates.

## Conclusion

In summary, using an fNIRS hyperscanning approach, we captured IBC changes in the inferior frontal cortex during and after emotional sharing. The trajectory of IBC may be explained from an *emotional co-regulation* perspective (Butler & Randall, 2013), including both emotional contagion and buffering. Fluctuations in IBC were associated with changes in mood for both sharers and listeners. These results suggest that IBC could provide a unique neural window into emotional sharing, increasing as a function of emotional processing and regulation. These findings, while requiring further consolidation, offer valuable insights into the dynamics of emotional sharing and their significance for fostering deeper interpersonal connections, particularly in close relationships (i.e., friendships). Moreover, the proposed method holds potential relevance for informing psychotherapeutic interventions by advancing understanding of the interpersonal neural processes that may support emotional co-regulation.

## Supplementary Information

Below is the link to the electronic supplementary material.

Supplementary file1 (DOCX 345 KB)

## Data Availability

De-identified data is available from the corresponding author upon reasonable request. The experiment was not preregistered.

## Code availability

Code is available from the corresponding author upon reasonable request.
