# Supplementary material for: Inter-brain coupling tracks emotional co-regulation
Source: Cogn Affect Behav Neurosci. 2026 May 23;26(5):2167–79. doi: 10.3758/s13415-026-01455-9 (PMC13615101; doi:10.3758/s13415-026-01455-9)
Supplement: Supplementary file 1 [file 13415_2026_1455_MOESM1_ESM.docx]

**Supplementary Materials**

**Motion energy analysis**

First, the motion energy analysis (MEA) algorithm was applied to two regions of interest (ROIs): ROI #1 covering the viewer’s whole body and ROI #2 covering the interviewer’s whole body. Continuous measures of movement were computed and extracted for each ROI. Second, the raw motion time series for both the viewer and the interviewer were preprocessed: signals were smoothed using a moving average window of 0.5 s; values exceeding the mean plus 10 times the standard deviation of the time series were identified as outliers and removed; the resulting series were then standardized. Third, the preprocessed data were analyzed using cross-correlation. For each dyad and condition, movement time series of the viewer and interviewer were cross-correlated using moving windows of 30 s to accommodate the non-stationary nature of movement behaviors. The maximum time lag was set to ±5 s in steps of 0.04 s, yielding 125 steps in each direction, consistent with previous studies to ensure comparability (Pan et al. 2021, 2022). Finally, movement synchrony was estimated from the window-averaged, Fisher’s z-transformed absolute cross-correlation coefficients, encompassing all 251 time lags (125 positive, 125 negative, and zero lag). Both positive and negative cross-correlations contributed positively to movement synchrony. Following prior work, an overall movement synchrony index was derived by averaging the cross-correlation coefficients across all time lags.

**Fig. S1.** Cluster-based permutation tests. The analysis captures the largest cluster (i.e., the summed *t* statistics within a contiguous supra-threshold cluster, threshold *P* < 0.05) as the frequencies and regions of interest for both sharing and post-sharing stages.

**Fig. S2**. The relationships between inter-brain coupling (IBC) and Profile of Mood States (POMS) scores in both sharers and listeners. △ = Post sharing minus sharing. Reported *P* values are uncorrected.

**References**

Pan Y, Dikker S, Zhu Y, Yang C, Hu Y, Goldstein P. 2022. Instructor-learner body coupling reflects instruction and learning. npj Sci Learn. 7:15.

Pan Y, Novembre G, Song B, Zhu Y, Hu Y. 2021. Dual brain stimulation enhances interpersonal learning through spontaneous movement synchrony. Soc Cogn Affect Neurosci. 16:210–221.
